# Supplementary figures and images for: Comparison of the dynamics of exoskeletal-assisted and unassisted locomotion in an FDA-approved lower extremity device: Controlled experiments and development of a subject-specific virtual simulator
Source: PLoS One. 2023 Feb 10;18(2):e0270078. doi: 10.1371/journal.pone.0270078 (PMC9916583; doi:10.1371/journal.pone.0270078)

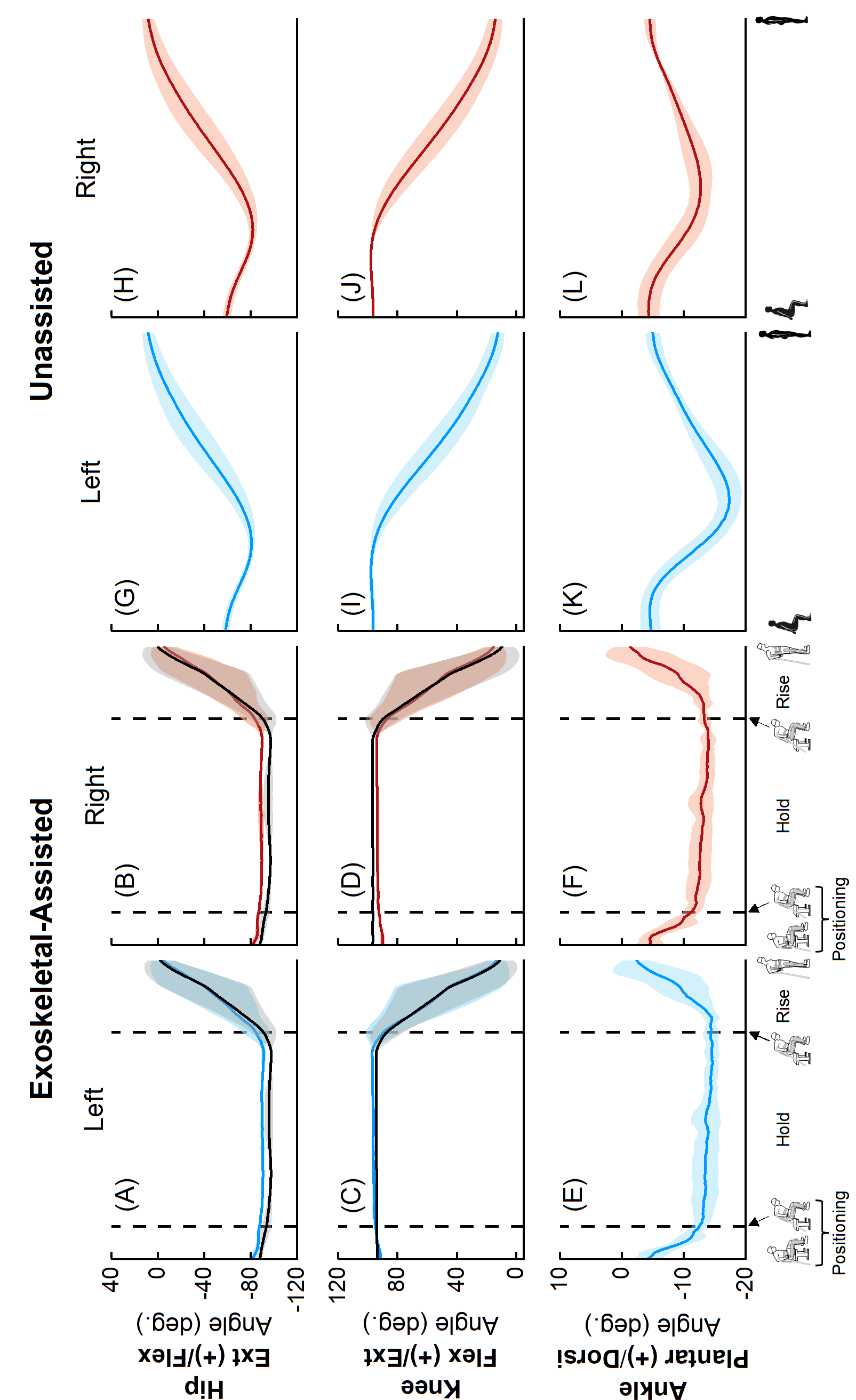

Supplement: S1 Fig — The offset in angles between the human (blue or red) and the robot (black) was due to different definitions of coordinate axes for each rigid body. (A-F) The first dashed vertical lines represent the transition from positioning phase (user gets in to position by flexing the torso forward and loading the crutches) to hold phase (leaned forward for 3 seconds). The second dashed vertical lines represent the beginning of the rise phase, with the hip and knee motors activating to complete the sit-to-stand maneuver. (TIF) [file pone.0270078.s001.tif]

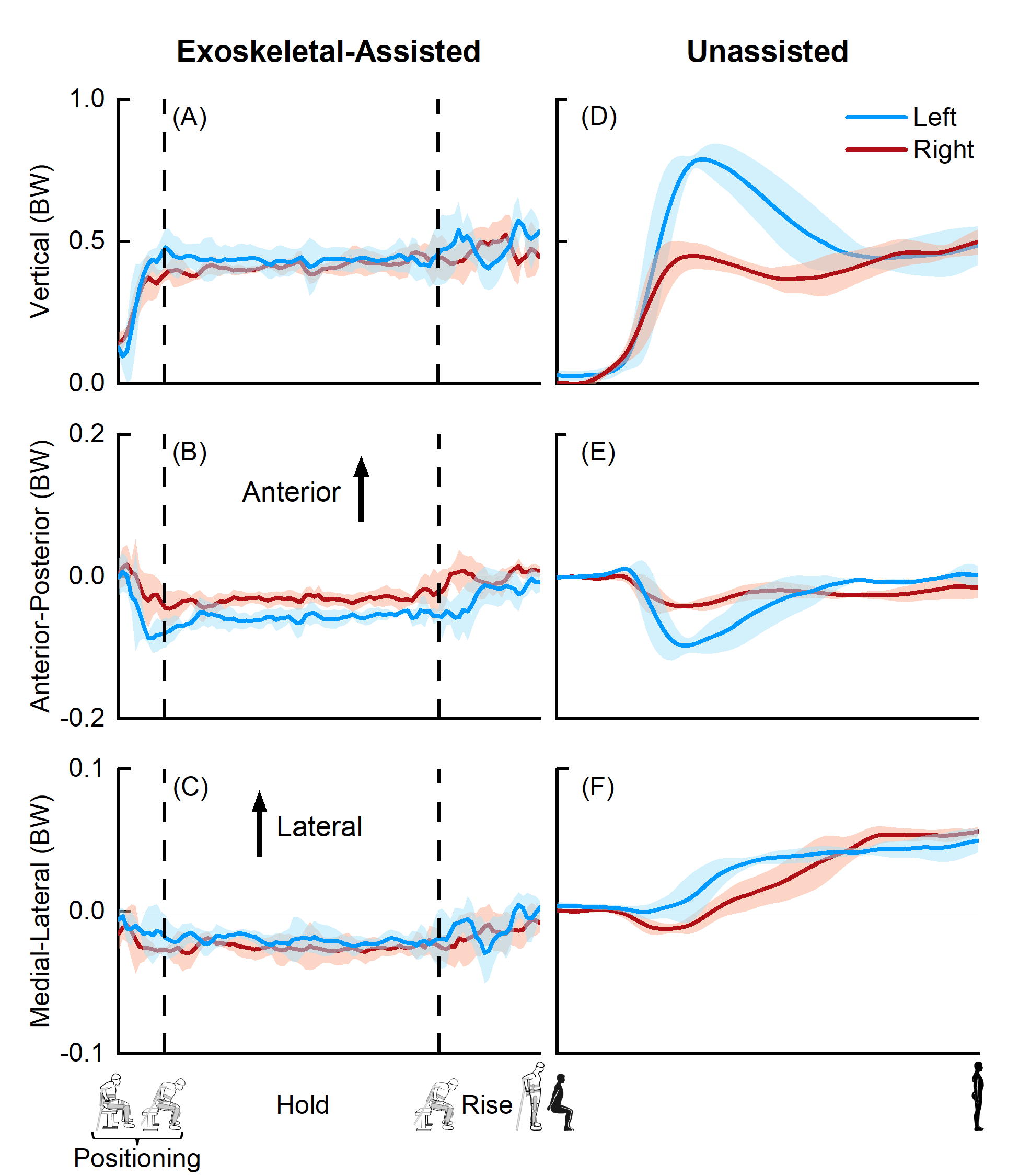

Supplement: S2 Fig — The ground reaction forces from exoskeletal-assisted locomotion were normalized to the combined weight of the participant and the exoskeleton; forces from unassisted locomotion were normalized to the participant’s body weight (BW). (A-C) The first dashed vertical lines represent the transition from positioning phase (user gets in to position by flexing the torso forward and loading the crutches) to hold phase (leaned forward for 3 seconds). The second dashed vertical lines represent the beginning of the rise phase, with the hip and knee motors activating to complete the sit-to-stand maneuver. A-P = Anterior-Posterior, M-L = Medial-Lateral. (TIF) [file pone.0270078.s002.tif]

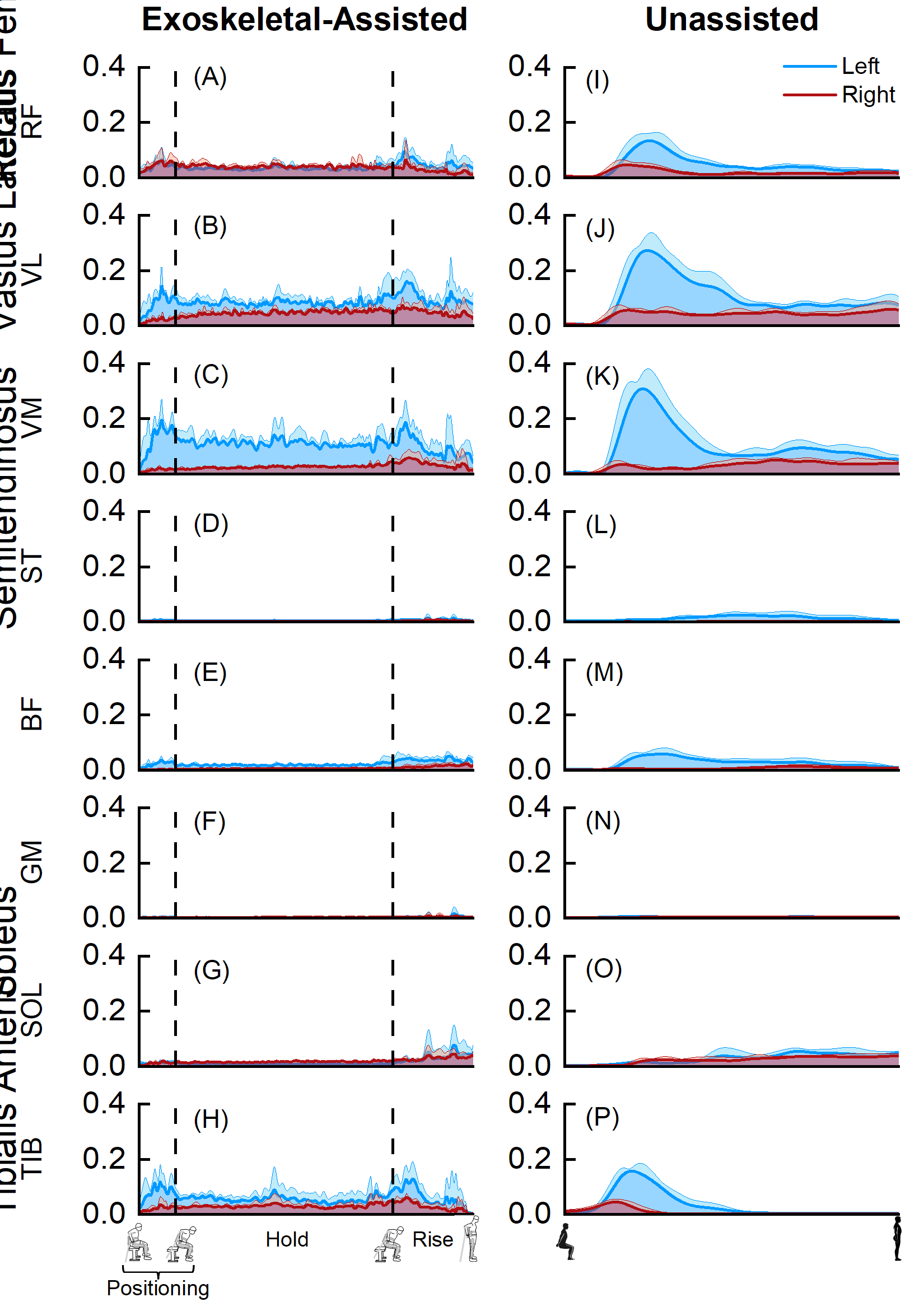

Supplement: S3 Fig — The EMG data were normalized using muscle-specific maximum voluntary contraction values. (A-H) The first dashed vertical lines represent the transition from positioning phase (user gets in to position by flexing the torso forward and loading the crutches) to hold phase (leaned forward for 3 seconds). The second dashed vertical lines represent the beginning of the rise phase, with the hip and knee motors activating to complete the sit-to-stand maneuver. RF = rectus femoris, VL = vastus lateralis, VM = vastus medialis, ST = semitendinosus, BF = biceps femoris, GM = gastrocnemius medialis, SOL = soleus, TIB = tibialis anterior. (TIF) [file pone.0270078.s003.tif]

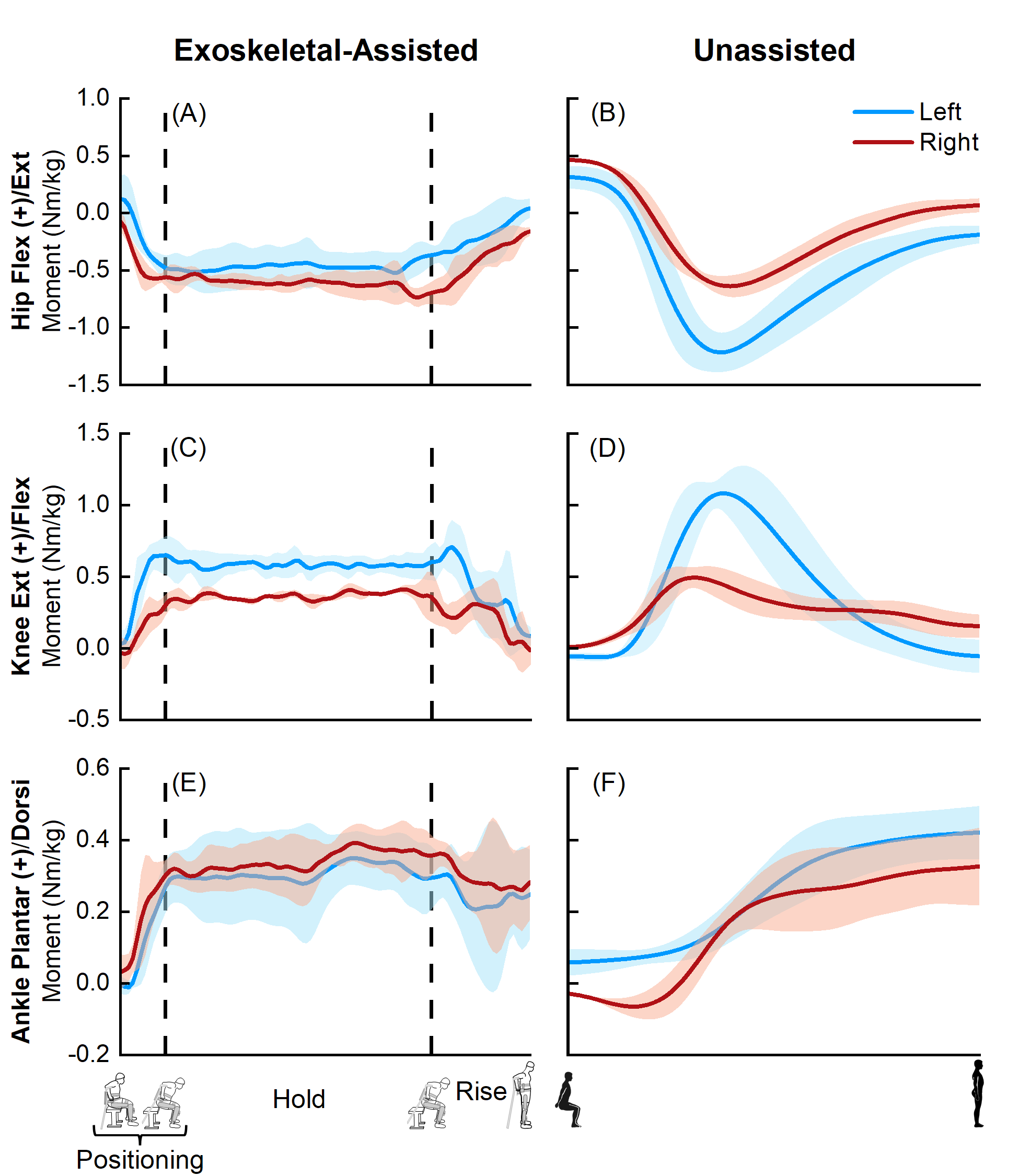

Supplement: S4 Fig — The joint moments from exoskeletal-assisted locomotion were normalized to the combined mass of the participant and the exoskeleton; joint moments from unassisted locomotion were normalized to the participant’s mass. (A-F) The first dashed vertical lines represent the transition from positioning phase (user gets in to position by flexing the torso forward and loading the crutches) to hold phase (leaned forward for 3 seconds). The second dashed vertical lines represent the beginning of the rise phase, with the hip and knee motors activating to complete the sit-to-stand maneuver. (TIF) [file pone.0270078.s004.tif]

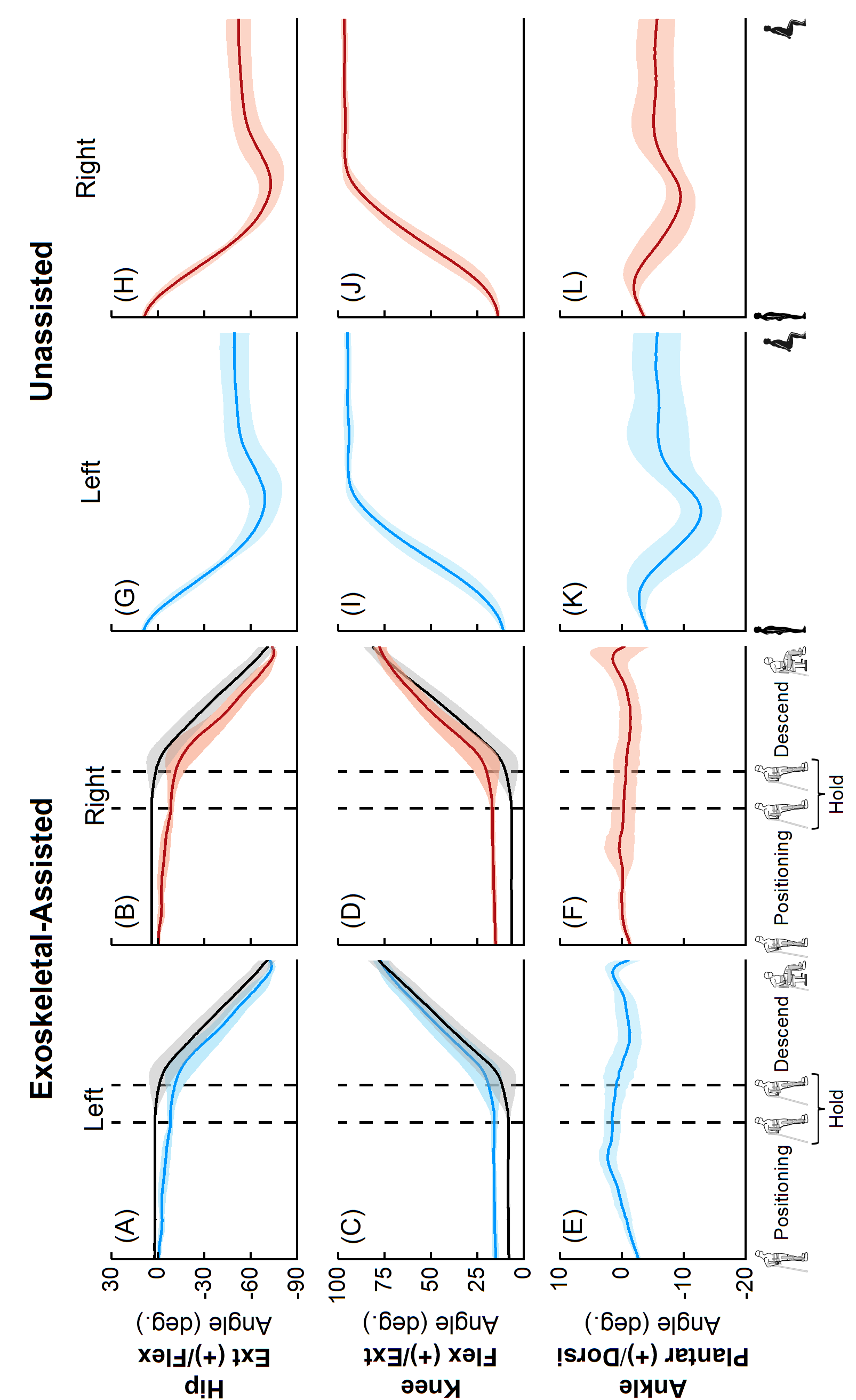

Supplement: S5 Fig — The offset in angles between the human (blue or red) and the robot (black) was due to different definitions of coordinate axes for each rigid body. (A-F) The first dashed vertical lines represent the transition from positioning phase (user gets in to position by leaning backwards and loading the crutches) to hold phase (leaned backward for 3 seconds). The second dashed vertical lines represent the beginning of the descend phase, with the hip and knee motors activating to complete the stand-to-sit maneuver. (TIF) [file pone.0270078.s005.tif]

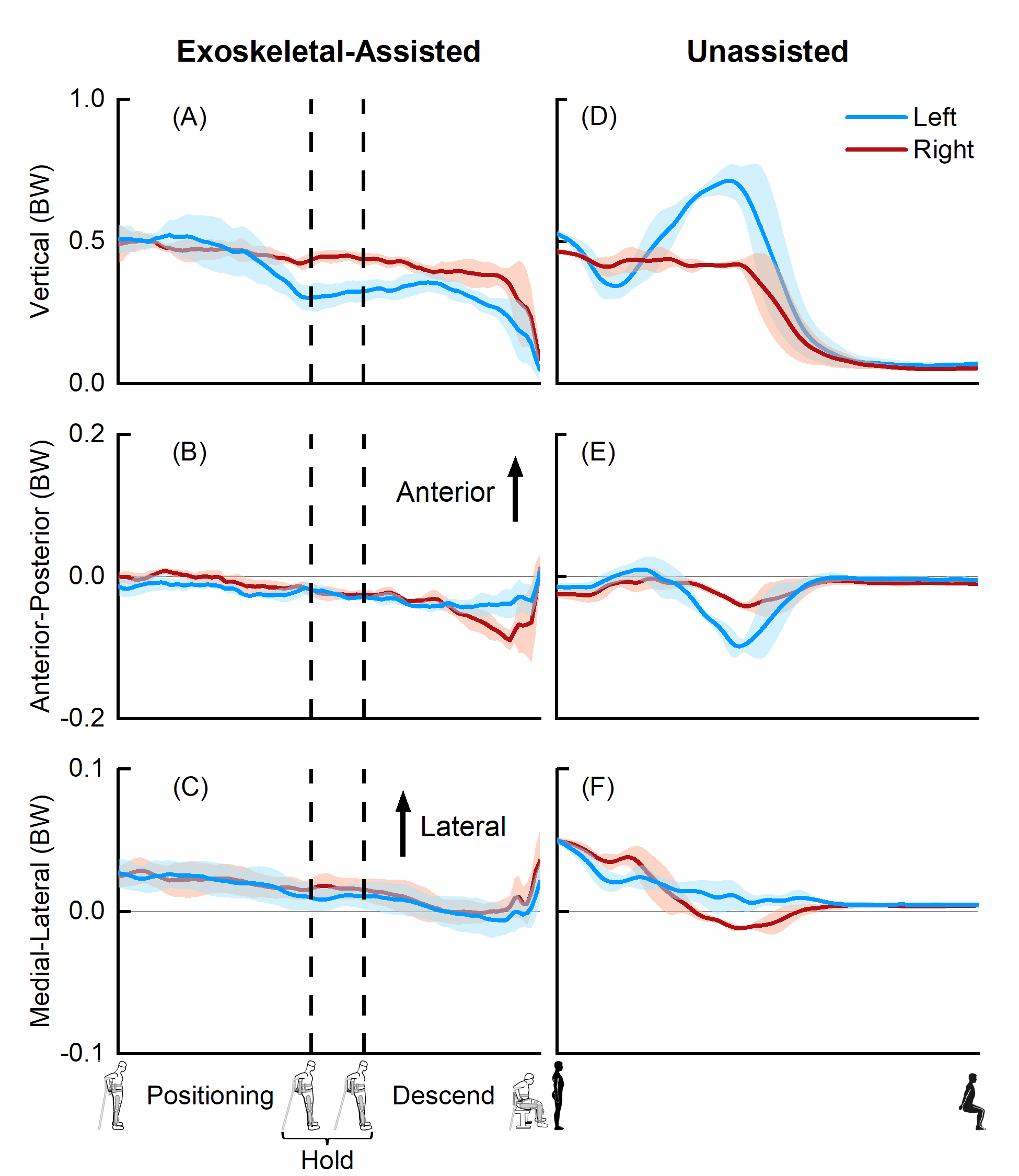

Supplement: S6 Fig — The ground reaction forces from exoskeletal-assisted locomotion were normalized to the combined weight of the participant and the exoskeleton; forces from unassisted locomotion were normalized to the participant’s body weight (BW). (A-C) The first dashed vertical lines represent the transition from positioning phase (user gets in to position by leaning backwards and loading the crutches) to hold phase (leaned backward for 3 seconds). The second dashed vertical lines represent the beginning of the descend phase, with the hip and knee motors activating to complete the stand-to-sit maneuver. A-P = Anterior-Posterior, M-L = Medial-Lateral. (TIF) [file pone.0270078.s006.tif]

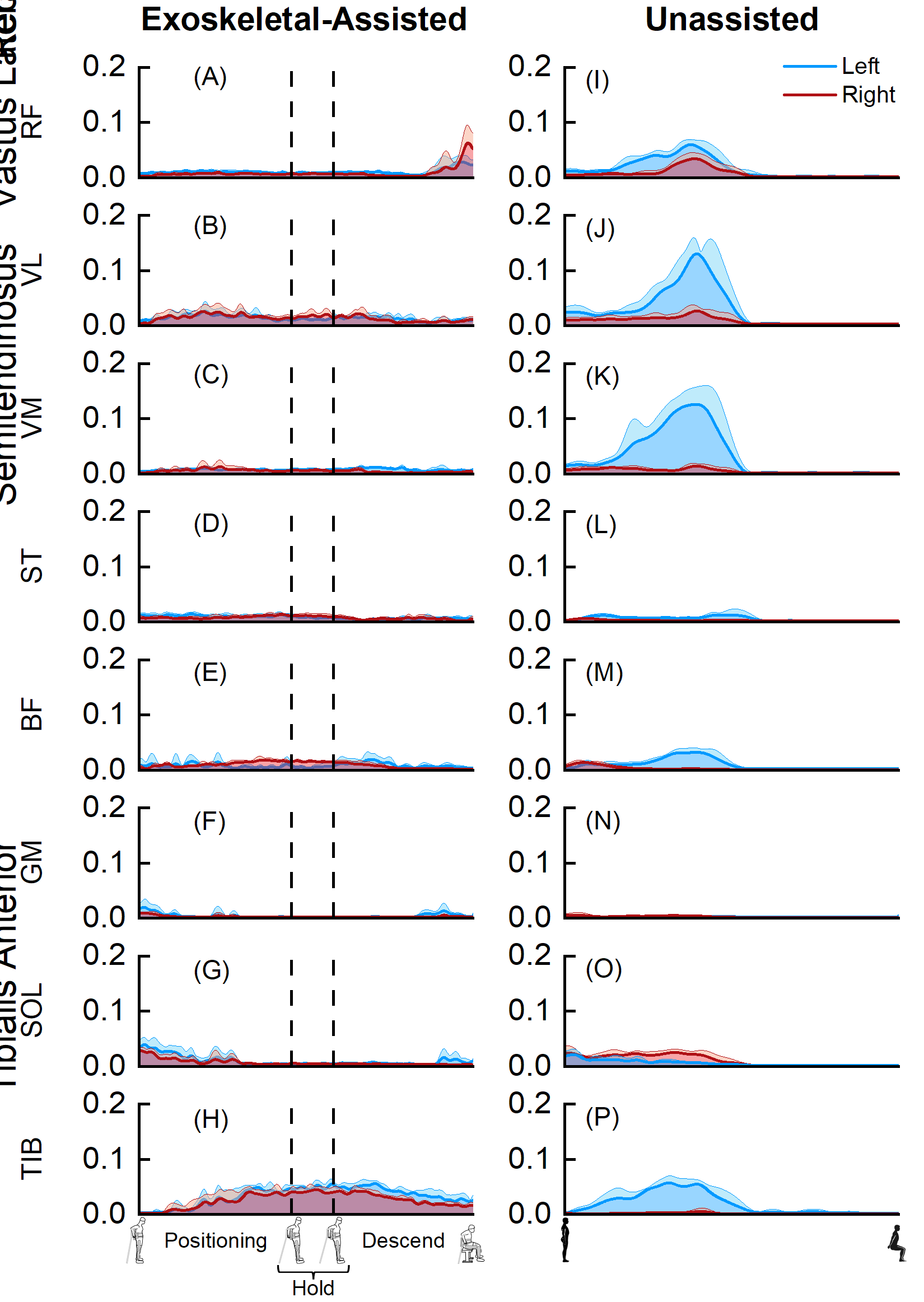

Supplement: S7 Fig — The EMG data were normalized using muscle-specific maximum voluntary contraction values. (A-H) The first dashed vertical lines represent the transition from positioning phase (user gets in to position by leaning backwards and loading the crutches) to hold phase (leaned backward for 3 seconds). The second dashed vertical lines represent the beginning of the descend phase, with the hip and knee motors activating to complete the stand-to-sit maneuver. RF = rectus femoris, VL = vastus lateralis, VM = vastus medialis, ST = semitendinosus, BF = biceps femoris, GM = gastrocnemius medialis, SOL = soleus, TIB = tibialis anterior. (TIF) [file pone.0270078.s007.tif]

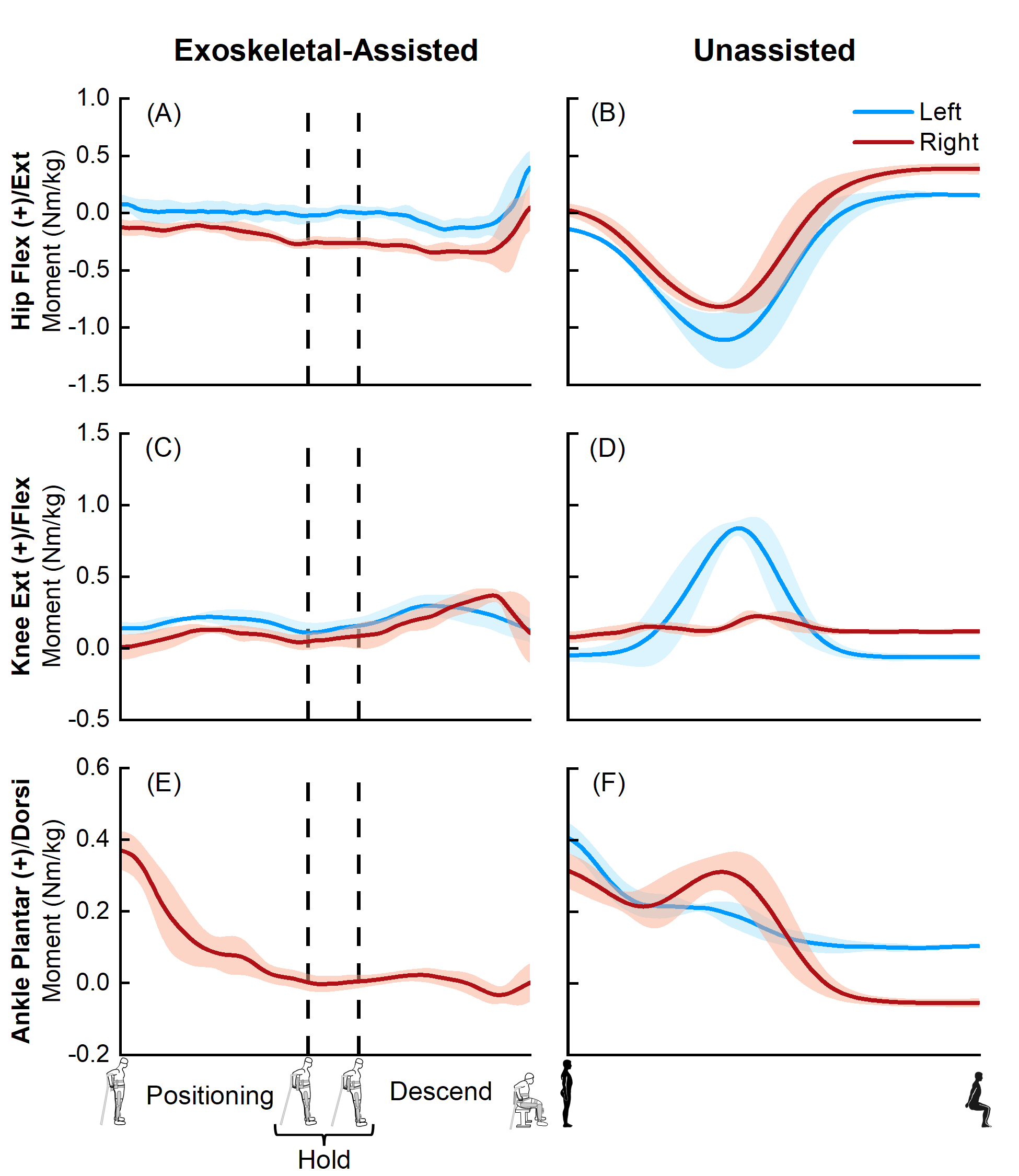

Supplement: S8 Fig — The joint moments from exoskeletal-assisted locomotion were normalized to the combined mass of the participant and the exoskeleton; joint moments from unassisted locomotion were normalized to the participant’s mass. (A-F) The first dashed vertical lines represent the transition from positioning phase (user gets in to position by leaning backwards and loading the crutches) to hold phase (leaned backward for 3 seconds). The second dashed vertical lines represent the beginning of the descend phase, with the hip and knee motors activating to complete the stand-to-sit maneuver. (TIF) [file pone.0270078.s008.tif]
