# Supplementary material for: Comparison of the dynamics of exoskeletal-assisted and unassisted locomotion in an FDA-approved lower extremity device: Controlled experiments and development of a subject-specific virtual simulator
Source: PLoS One. 2023 Feb 10;18(2):e0270078. doi: 10.1371/journal.pone.0270078 (PMC9916583; doi:10.1371/journal.pone.0270078)
Supplement: Attachment — Submitted filename: Response to Reviewers.pdf [file pone.0270078.s009.pdf]

Response to Reviewers

We thank the editor and the reviewers for their thorough review of our work. Responding to the reviewers' comments has improved this manuscript. The specific responses to the comments are described below, and the changes are highlighted in the revised manuscript. Please note that the sentences in italics are reviewer comments.

Journal Requirements:

When submitting your revision, we need you to address these additional requirements.
1. Please ensure that your manuscript meets PLOS ONE's style requirements, including those for file naming. The PLOSONE style templates can be found at
https://journals.plos.org/plosone/s/file?id=wjVg/PLOSOne_formatting_sample_main_body.pdf
and
https://journals.plos.org/plosone/s/file?id=ba62/PLOSOne_formatting_sample_title_authors_affiliations.pdf

Authors' Response: We have modified the manuscript to comply with style requirements for PLOS ONE.

2. Thank you for providing the following information in your Ethics Statement: "The Institutional Review Board at New Jersey Institute of Technology approved this study. The approval number is 2008001531R001." We ask that you also amend your Methods section to include this statement.

Authors' Response: We added the requested sentence in the Methods section.

3. Thank you for stating in your Funding Statement:
"WAB and SP received support from the Department of Veterans Affairs (https://www.va.gov/) grant VA RR&D # 1 I01RX003561-01A2. The funders had no role in study design, data collection and analysis, decision to publish, or preparation of the manuscript."
*Please provide an amended statement that declares *all* the funding or sources of support (whether external or internal to your organization) received during this study, as detailed online in our guide for authors at http://journals.plos.org/plosone/s/submit-now. Please also include the statement "There was no additional external funding received for this study." in your updated Funding Statement.*
Please include your amended Funding Statement within your cover letter. We will change the online submission form on your behalf.

Authors' Response: We included an amended Funding Statement in our cover letter, as requested.

Reviewers' Comments:

5. Review Comments to the Author

Reviewer #1

This is a well-written paper that describes an experiment and simulator with one subject performing walking and sit-to-stand motions with and without an exoskeleton. I believe the manuscript is eventually worthy of publication, though I have a number of major and minor comments.

Authors' Response: We thank the reviewer for their positive feedback.

MAJOR REVISION

1. The authors repeatedly emphasize that existing studies have not been performed on an FDA-approved exoskeleton, and that the novelty of the work is usage with an FDA-approved exoskeleton. However, it is unclear why the FDA approval represents a major difference. It seems like existing studies have achieved similar results with less popular exoskeletons, and most of the approaches used in existing studies can be approved to other exoskeletons with relatively little modification. The authors should better explain why the study is more than simply applying existing methods to a more popular exoskeleton.

Authors' Response: We thank the reviewer for the opportunity to clarify why we restricted our work to an FDA-approved exoskeleton device. It is important to study the dynamics of the human-robot system during exoskeletal-assisted locomotion (EAL) in FDA-approved devices because individuals with neurological disorders only have access to FDA-approved devices. Currently, there is a disconnect between the exoskeletons used by patients and exoskeletons used to study human-robot interaction. Our study is a first step to address this disconnect. We have added several sentences in Introduction to highlight the importance of studying the dynamics of exoskeleton-assisted locomotion in FDA-approved devices.

Next, prior studies with FDA-approved exoskeletons have quantified joint kinematics, EMG, foot reaction forces, and ground reaction forces. To the best of our knowledge, no prior study has reported joint moments of the human-robot system during EAL in an FDA-approved device. We have clarified this gap in knowledge in Introduction section.

2. Related to the above: the Discussion acknowledges that several studies have already been done with the ReWalk exoskeleton (which the authors are using). The authors should better characterize how their work advances previous ReWalk work rather than simply saying their results are in line with previous ReWalk results.

Authors' Response: We have added a paragraph in the Discussion section to highlight how our work advances previous work performed with the ReWalk exoskeleton, as requested.

3. Why did the authors recruit only a single subject for a single session? It seems like there would be significant intersubject variability that would both make it difficult to compare exoskeleton vs.

no-exoskeleton conditions as well as make it difficult to evaluate performance of a simulation model. Additionally, since this was an able-bodied subject who only participated for a few sessions, it seems like the authors could have fairly easily obtained a larger sample size.

Authors' Response: We agree with the Reviewer that there will be some inter-subject variability and additional participants will be required to validate the findings of this study. We respectfully disagree that adding additional participants to our study would be easy. To this point, conducting the controlled experiments and developing the subject-specific virtual simulators of walking, sit-to-stand, and stand-to-sit maneuvers with and without the exoskeleton are extremely laborious and time-consuming tasks. We currently are actively recruiting 7 able-bodied and 10 spinal cord injured participants to extend this work and anticipate that it will take at least a couple of years to recruit, study, and complete the analyses on these participants.

To clarify, the goal of this manuscript is to report our novel framework combining controlled experiments with computational modeling to build subject-specific virtual simulators of EAL that reproduces walking, sit-to-stand, and stand-to-sit maneuvers. Developing this framework represents substantial effort. We have described our methods in sufficient detail for the research community to reproduce our work. In addition, we will make all empirical data from this study freely available to the research community. There is benefit to publishing our dataset now so the modeling community can start building on our work to address a broad range of research questions. We have added several sentences in the section that addresses study limitations to clarify this point.

4. The simulation model does not appear to be extensively validated - the authors show that it can fairly accurately reproduce actual trajectories, but this is done by training and testing on the same subject in the same session. Is this sufficient for a real simulator? I would have expected it to be validated by examining predictive ability in different situations. And when the authors say that the simulator reproduced actual trajectories "within acceptable tolerances", what is an acceptable tolerance?

Authors' Response: We agree that we have not yet tested the predictive capability of our virtual simulators. As we have stated previously in our response, the goal of this study was to develop the framework to build subject-specific virtual simulators of EAL. Testing the predictive capability of these virtual simulators will be a component of our future work. We invite other investigators to use our dataset to test the predictive capability of our virtual simulators, or to build their own virtual simulators from our empirical data. We have clarified in the Abstract that this work provides a foundation for parametric studies to characterize the effects of human and robot design variables, and predictive modeling to optimize human-robot interaction during EAL. We have also added a few sentences at the end of the Discussion section encouraging other investigators to build on our work.

Next, acceptable tolerance is less than 2 cm average RMS error between experiment and simulator markers, per OpenSim's best practices, which has been added with a sentence and two references in the Results section.

MINOR REVISION

1. Is it really necessary to list inclusion/exclusion criteria when only a single participant was recruited? Authors list things like pregnancy and lactation being exclusion criteria, which seems irrelevant for a male participant. They also list minimum and maximum weight, which again seems irrelevant since a single participant was recruited and his weight is known.

Authors' Response: We thank the reviewer for this comment and in response have removed the inclusion/exclusion criteria from the manuscript.

2. Could the authors briefly describe how maximum voluntary contraction protocols were performed?

Authors' Response: We have added several sentences in the Methods section to describe our maximum voluntary contraction trials.

3. Why were there 4 trials of exoskeleton-assisted sit-to-stand and 5 trials of exoskeleton-unassisted sit-to-stand? Why not 5 for both?

Authors' Response: We collected 10 trials each of walking, sit-to-stand, and stand-to-sit maneuvers, but only successful trials were included for further analyses. We have clarified in the Methods section the criteria for a successful trial for each maneuver. Meeting these criteria resulted in different numbers of trials for the different maneuvers. Based on these criteria, we obtained 6 successful trials each of exoskeletal-assisted and unassisted walking, 4 and 5 successful trials of exoskeletal-assisted and unassisted sit-to-stand maneuvers, respectively, and 5 successful trials each of exoskeletal-assisted and unassisted stand-to-sit maneuvers.

Reviewer #2

The manuscript presents a dataset collected on a healthy subject performing exoskeleton-assisted vs unassisted locomotion using an FDA-approved exoskeleton (ReWalk P6.0), and a subject-specific virtual simulator implemented using OpenSim environment, to compute joint kinematics (from motion capture and simulation) and moments (from simulation) for hip, knee and ankle in assisted vs unassisted conditions. In addition, EMG data from 8 lower-limb muscles were recorded, as well as ground reaction forces using force plates.

The main novelty claimed by the authors is the availability of such a dataset and simulation that includes an FDA-approved exoskeleton comparing assisted and unassisted locomotion. Nevertheless, whereas the need for having such a dataset and simulation is well explained in the manuscript, it is not clear which is the added value of including an FDA-approved exoskeleton, with respect to other examples (cited by the authors in the manuscript) using different exoskeletons. In my opinion, this alone cannot be the main claim of the manuscript given that: no novel methods are presented; different exoskeletons might result in different human-robot interaction when assistance is delivered; other similar data in literature could be used for the simulator if the benefit would be the possibility to "conduct rapid design-phase evaluations to accelerate device refinements" (Discussion).

Authors' Response: We thank the reviewer for this comment and have addressed the importance of studying the dynamics of exoskeleton-assisted locomotion in FDA-approved devices in Reviewer 1's first major revision comment. To briefly reiterate our prior response, it is important to study the dynamics of the human-robot system during EAL in FDA-approved devices because individuals with neurological disorders only have access to FDA-approved devices. Currently, there is a disconnect between the exoskeletons used by patients and exoskeletons used to study human-robot interaction. Our study is a first step to address this disconnect. We have added several sentences in the Introduction section to highlight the importance of studying the dynamics of exoskeleton-assisted locomotion in FDA-approved devices.

Next, our framework that combines controlled experiments with computational modeling to build subject-specific virtual simulators of EAL is novel. No prior study has simulated EAL in any FDA-approved exoskeleton. The minimum empirical dataset we will share as part of this publication is novel. There is no such dataset currently available to the research community, which explains why the computational modeling community has developed virtual simulators based on idealized or imaginary exoskeletons (Bianco et al., 2022; Dembia et al., 2017; Khamar et al., 2019; Nguyen et al., 2019; Uchida et al., 2016; Zhu et al., 2015). Furthermore, we have added two paragraphs in the Discussion section to highlight how our work advances previous work performed with the ReWalk and other FDA-approved exoskeletons.

The authors have stated clearly the limitations of the study, including having no participants with altered gait patterns (e.g., spinal cord) and not separating human and robotic dynamics in the virtual simulator (one aspect that was described in the Introduction as particularly important and not very well explored in the state of the art). Exploring further more at least one of these limitations would be important to justify the significance of the manuscript. Indeed, in the current state, comparison with other studies involving impaired subjects (e.g., in the discussion) has a questionable significance.

Authors' Response: We thank the reviewer for this comment and have modified our text to provide a more detailed discussion of the limitation of including a single able-bodied participant in this study. Next, we have modified the text in the Discussion section to clarify how our work advances previous work done with the ReWalk and other FDA-approved exoskeletons.

Other comments:
- Kinematics' results are reported in the Figures without the offset, but absolute values of data without removing the offset are comparing in the manuscript. How should these differences be interpreted? Comparing and presenting directly data without offset should be more straightforward.

Authors' Response: This comment is somewhat unclear to the authors. However, in our attempt to clarify, our segment coordinate frames were obtained with reference to a single global coordinate frame. The initial values of the joint kinematics from the different segment coordinate frames were based on this single global coordinate frame. This provided a direct comparison of joint kinematics with and without the exoskeleton. For example, using this approach we clearly see that the participant's hips were more flexed during exoskeletal-assisted walking compared to unassisted walking (Figs 2A and 2B). If we were to remove the offset angles between the different segment coordinate axes, this would mask many of the kinematic differences that are of interest when

analyzing locomotion with and without the exoskeleton. In our results, we have only subtracted the offset angles to calculate the average absolute differences in kinematics between the human and the robot during exoskeletal-assisted walking (Figs 2A-2D). To clarify the rationale for the approach taken, text in the Results section has been appropriately edited.

- The standard deviation values of the RMS errors between experiments and simulation should be reported together with the mean values.

<u>Authors' Response:</u> We have included the standard deviation values of the RMS errors between experiment and simulator markers, as requested.
