# Supplementary material for: Comparison of the dynamics of exoskeletal-assisted and unassisted locomotion in an FDA-approved lower extremity device: Controlled experiments and development of a subject-specific virtual simulator
Source: PLoS One. 2023 Feb 10;18(2):e0270078. doi: 10.1371/journal.pone.0270078 (PMC9916583; doi:10.1371/journal.pone.0270078)
Supplement: Attachment — Submitted filename: Response to Reviewers.pdf [file pone.0270078.s010.pdf]

Response to Reviewers

We thank the Editor and the Reviewers for their thorough review of our work. Responding to the Reviewers' comments has improved this manuscript. The specific responses to the comments are described below, and the changes are highlighted in the revised manuscript. Please note that the sentences in italics are reviewer comments.

Journal Requirements:

Please review your reference list to ensure that it is complete and correct. If you have cited papers that have been retracted, please include the rationale for doing so in the manuscript text, or remove these references and replace them with relevant current references. Any changes to the reference list should be mentioned in the rebuttal letter that accompanies your revised manuscript. If you need to cite a retracted article, indicate the article's retracted status in the References list and also include a citation and full reference for the retraction notice.

Authors' Response: We have checked our reference list to ensure it is complete and correct. We are not aware of any paper cited that has been retracted. There are no changes to the reference list in this round of review.

Additional Editor Comments:

Please pay attention to the issues raised by the reviewer 1.

Authors' Response: We thank the Editor and the Reviewers for their comments. We have addressed all the issues raised by both the reviewers to the best of our ability.

Reviewers' Comments:

6. Review Comments to the Author

Reviewer #1

The authors have partially addressed my comments from the first round of review, and I believe the paper is now somewhat closer to publication. However, I have several remaining comments and thus recommend another round of major revisions.

Authors' Response: We thank the Reviewer for their positive feedback.

MAJOR ISSUES

1. The authors repeatedly emphasize that the novel dataset collected during the study would be of value to researchers. However, as far as I can tell, they have not made the dataset available, and

present conflicting statements in the PLOS ONE submission system. The Data Availability box states "Yes - all data are fully available without restriction", but the "Describe where the data may be found in full sentences" box then simply states "All empirical data from this study will be freely available." The Discussion also states that all data "will be" fully available. If the novel dataset is part of the value of the manuscript, the authors should make it available during the review process. If that is not possible for some exceptional reason, the authors should at least provide some indication of how the data will be made available so that the approach can be evaluated.

Authors' Response: We have clarified in the text that the dataset will be made available through an online repository. This dataset will be made available upon acceptance of this manuscript, consistent with PLOS ONE's policies and advice of the Academic Editor.

2. The authors state that, to their knowledge, no previous study has reported joint moments. I would appreciate a brief explanation of how difficult it is to obtain these joint moments and what the usefulness of joint moments is relative to other data that was already previously done. This would help evaluate the degree of methodological novelty.

Authors' Response: To clarify, no prior study has reported joint moments in an FDA-approved device. We have added several sentences in the Introduction to explain the importance of joint moments and the difficulty in quantifying joint moments.

3. I agree that patients have access only to FDA-approved devices, but at the same time, the authors claim that the goal of their work is to "report our novel framework combining controlled experiments with computational modeling to build subject-specific virtual simulators". A novel framework should be applicable to many exoskeletons, not just the specific FDA-approved one, and it is thus still not convincing why the authors emphasize FDA approval so much. Is the novelty that this is the first empirical dataset of this scope with an FDA-approved device?

Authors' Response: We have clarified in the last paragraph of Discussion that our framework is applicable to exoskeletons that are not FDA-approved.

We have explained in the prior round of revision the importance of studying exoskeletal-assisted locomotion (EAL) in FDA-approved devices. The Reviewer concurs with our explanation that patients with neurological disorders only have access to FDA-approved devices. This is the primary reason to study EAL in FDA-approved devices. This is currently not happening because of the lack of empirical data. We are providing these empirical data to engage the research community to study EAL in FDA-approved devices. We are confident this will directly benefit patients with neurological disorders.

Yes, the Reviewer is correct that part of the novelty of this study is that it will provide the first empirical dataset with an FDA-approved device. We have highlighted this in two places in the Discussion.

4. The authors' justification for why only 1 subject is possible is that it would take years to conduct a study with ~20 participants. If development of virtual simulators for specific individuals is so time-consuming (seemingly on the scale of months / participant), the authors should better justify the expected practical value of such simulators. For real patients, economic and logistical issues would likely not allow months of time to be devoted to tuning a simulator to each person.

Authors' Response: There is potentially immense value to virtual simulators of EAL developed from controlled experiments. We are doing the hard work to build these virtual simulators from controlled experiments in our lab. Once they are developed, our lab and others in the research community should be able to perform a broad range of computational studies in an efficient manner. Real patients will not be required for these computational studies.

MINOR ISSUES

1. The authors' justification for why it is important to study the dynamics of the human-robot system during EAL in FDA-approved devices appears to have been awkwardly dropped into the Introduction without much effort to make it "flow" well with the rest of the text. Would recommend better integrating with previously existing text.

Authors' Response: We have added the justification as a separate paragraph in an effort to improve the flow of the text. We are open to suggestions to improve the flow of the text.

2. Lines 171-172: "A generic musculoskeletal [55] was scaled to the participant's anthropometry." A noun seems to be missing here.

Authors' Response: Thank you for pointing this out. We have added "model" in the text.

3. The authors state that they conducted 10 trials for each activity and then analyzed only the successful trials, leading to different numbers of trials in each activity. Why did the authors alternatively not repeat trials until a certain number of successful trials was obtained? Is it not possible to check for missing data within the session?

Authors' Response: Unfortunately, checking for missing data in the exoskeleton's encoder in real time is currently not possible. We would have to reset the exoskeleton every time we access the encoder for checking the data; this would add substantial time to our experiments with a real human subject.

4. The Introduction says that the new dataset is novel because it includes joint moments, which had not been previously available. The Discussion then says that the new dataset is novel because it includes joint moments and EMG. If the EMG is also novel, I recommend mentioning that in the Introduction as well.

Authors' Response: In the Introduction, we list four studies that have reported EMG from EAL in the Ekso and Indego, both FDA-approved exoskeletons. The Discussion section that the Reviewer is referring to clarifies how our study advances previous work done in the ReWalk device; this section was added in response to Reviewer 1's request in the previous round. For the ReWalk device, no prior study has reported EMG or joint moments, as we have highlighted in our Discussion.

Reviewer #2:

The authors have addressed the Reviewers' comments and the contribution of the manuscript is more clear.

Authors' Response: We thank the Reviewer for their positive feedback.

I would suggest to carefully check the new text that was included to avoid repetitions (i.e., the sentence "we will make all empirical data from this study freely available to the research community" is repeated twice in the Discussion). Regarding this point, it would be more sound to write in the manuscript that the dataset is already available (instead of "will be available").

Authors' Response: We thank the Reviewer for this comment. We have removed repetitions from the manuscript.

Our dataset will be available prior to the publication of this manuscript. We will upload our dataset to an online repository as soon as this manuscript is accepted, consistent with PLOS ONE's policies and advice of the Academic Editor.
